# A cross-sectional study: caregiver burden and related determinants of adult patients with β-thalassemia major in mainland China

**DOI:** 10.1186/s12912-024-01826-y

**Published:** 2024-03-04

**Authors:** Runqi Zhang, Shuo Zhang, Jing Ming, Jing Xie, Baoguo Liu, Weihang Jiang, Yingjie Fu, Xuemei Zhen, Xiaojie Sun

**Affiliations:** 1 Centre for Health Management and Policy Research, School of Public Health, Cheeloo College of Medicine, Shandong University, Jinan, Shandong 250012 China; 2 NHC Key Lab of Health Economics and Policy Research (Shandong University), Jinan, Shandong 250012 China; 3 Shandong Public Health Clinical Center, Jinan, 250100 China; 4 New Sunshine Charity Foundation, Beijing, 100097 China

**Keywords:** Caregiver burden, β-thalassemia major, Adult patient, Zarit burden interview

## Abstract

**Background:**

The informal caregivers of adult patients with β-thalassemia major (β-TM) bear not only physical but also emotional and economic pressures of providing care. This study is the first to evaluate the caregiver burden by Zarit Burden Interview (ZBI) of adult patients with β-TM in mainland China and to identify predictors of caregiver burden.

**Methods:**

In this cross-sectional study, we conducted an online survey with snowball sampling covering seven provinces between September 1, 2021, and January 31, 2022, of patients aged ≥ 18 years with β-TM and their informal caregivers. Caregiver burden was assessed using the ZBI. Data on patient demographics, disease and therapy characteristics, and informal caregivers’ demographic characteristics were collected and analysed using independent t-tests, analysis of variance, Spearman’s correlation and multiple linear regression.

**Results:**

Of 75 included patients, more than half (50.7%) were male. The mean patient age was 24.69 ± 5.59 years. The mean age of the informal caregivers was 50.60 ± 9.16 years, with women (74.7%) being predominant. The ZBI score was 38.00 ± 17.02. Multiple linear regression analysis showed that patients with interrupted blood transfusion therapy and informal caregivers required to care of others were positively associated with caregiver burden (*p* < 0.05). Age of informal caregivers were borderline significant positively associated with caregiver burden (*p* < 0.1). Married informal caregivers were negatively associated with caregiver burden (*p* < 0.05).

**Conclusions:**

The informal caregivers of adult patients with β-TM in mainland China experienced a moderate-to-severe level of caregiving burden. The caregiver burden was higher in patients with a history of interrupted blood transfusion therapy or in informal caregivers who were older or needed to care for others. Additionally, married informal caregivers experienced lower burdens compared to non-married informal caregivers. These findings provide a reference to identify informal caregivers with higher burdens among patients with β-TM.

## Background

Thalassemia is a group of autosomal recessive genetic diseases caused by mutations in globin. There are two distinct forms of thalassemia according to the type of hemoglobin: α and β [1]. Patients with β-thalassemia can be divided into minor, intermediate, and major groups, based on the underlying genotype and modifier mutations [2]. Thereinto, β-thalassemia major (β-TM) is the most serious form, which often manifests in infancy, and can experience hemolytic anemia and hepatosplenomegaly [3]. β-thalassemia itself has high mortality and serious complications, becoming a global concern [4]. Approximately 1.5% (80–90 million people) individuals worldwide are carriers of β-thalassemia [5]. Moreover, worldwide, the prevalence of thalassemia is higher in Mediterranean countries, the Middle East, the Indian subcontinent, and South and Southeast Asia [6]. The nationwide prevalence of β-thalassemia in mainland China was roughly estimated at 0.66%, and up to 2.21% in high-incidence provinces (e.g., Guangdong, Guangxi, and Fujian) [7].

Patients with β-TM require lifelong regular blood transfusions and iron chelation therapy to survive. Iron overload caused by lifelong anemia and blood transfusions can lead to various complications (e.g., organomegaly and cardiac disease) that cause irreversible damage to multiple organs in the body and ultimately reduce life expectancy in these patients [8]. In Greece, 89% of patients with β-TM in Greece lived beyond the age of 40 years, while 80% of patients in the United Kingdom lived beyond the age of 45 years [9]; however, the highest life expectancy of patients with β-TM in Guangxi, China, was only 28 years [10]. Compared to children with β-TM, the quality of life of adult patients is significantly lower due to multiple organ dysfunction, chronic pain, and loss of physical fitness [11, 12].

Caregiver burden was defined by Montgomery et al. [13] as the perceived impact of caregiving tasks on caregivers’ emotions and on their resources [14]. Given the Chinese tradition, caregivers are mostly family members, especially parents. These family members act as “informal caregivers”, which were defined as unpaid family members, friends or neighbors who provided free care to the patient [15]. Due to poor quality of life, adult patients with β-thalassemia rely heavily on their informal caregivers, resulting in a severe caregiving burden [16]. Firstly, frequent blood transfusion therapy is a difficult and exhausting process, as the informal caregivers must accompany the patient through treatment, which reportedly results in feelings of helplessness and hopelessness among informal caregivers of patients with thalassemia. Therefore, informal caregivers must bear a heavy physical burden and psychological pressure during the process [17]. Secondly, due to frequent hospital visits, high medical expenses, and decreased life expectancy of patients, most informal caregivers of adult patients with β-TM bear great financial burdens [18]. Finally, informal caregivers need to frequently assist patients in their activities of daily living, administer medications, prepare meals, provide health care, and provide emotional support [19]. As a result, the responsibilities of parents of adult children with β-TM are diverse and affect various aspects throughout their lives [20].

Previous international studies have described the burdens and pressures experienced by most caregivers of patients with thalassemia, which involved the physical and mental health of these caregivers [21, 22]. For example, a study in India showed that the caregivers of children with thalassemia not only bore the heavy responsibility of care, but also experienced economic and emotional pressures [23]. However, these studies have mainly paid attention to pediatric patients with thalassemia, with very few studies focusing on caregiver burden of adult patients with β-thalassemia.

In mainland China, existed studies on the caregiver burden of patients with thalassemia have paid more attention to the child patients, while for the caregiver burden of adult patients there was still a research gap. In this study, we conducted a first nationwide survey on the caregiver burden of adult patients with β-TM, focusing on the high-incidence areas in mainland China, and for the first time examined the determinants of caregiver burden in these patients.

## Methods

### Sampling

This cross-sectional study conducted an online survey from September 1, 2021 to January 31, 2022 through “questionnaire star (https://www.wjx.cn)”, a platform offering respondents the ability to complete questionnaires online because an on-site survey was not feasible during the coronavirus disease 2019 (COVID-19) pandemic. On the one hand, complete medical information on patients was difficult to collect, considering the lack of epidemiological parameters nationwide, and the prevalence of thalassemia in different provinces is unclear. On the other hand, enough participants were difficult to seek out, because the patients’ visiting medical institution was not fixed due to the unstable supply of blood and iron chelation, as well as lifetime or irregular therapy. This study used snowball sampling covering seven provinces with a relatively high prevalence of thalassemia namely Guangdong Province and Guangxi Zhuang Autonomous Region, Fujian Province, Jiangsu Province, Jiangxi Province, Hunan Province, and Xinjiang Uygur Autonomous Region. Snowball sampling is a method of finding respondents in a sparse population by first randomly selecting a group of respondents, interviewing those respondents and then engaging them to deliver some other target group, and cycling through the process. In the study, patients were recruited through the website of the Beijing New Sunshine Charity Foundation and the Thalassemia Mutual Aid WeChat Group, and patient recommendations by doctors from representative medical institutions.

**Fig. 1:**
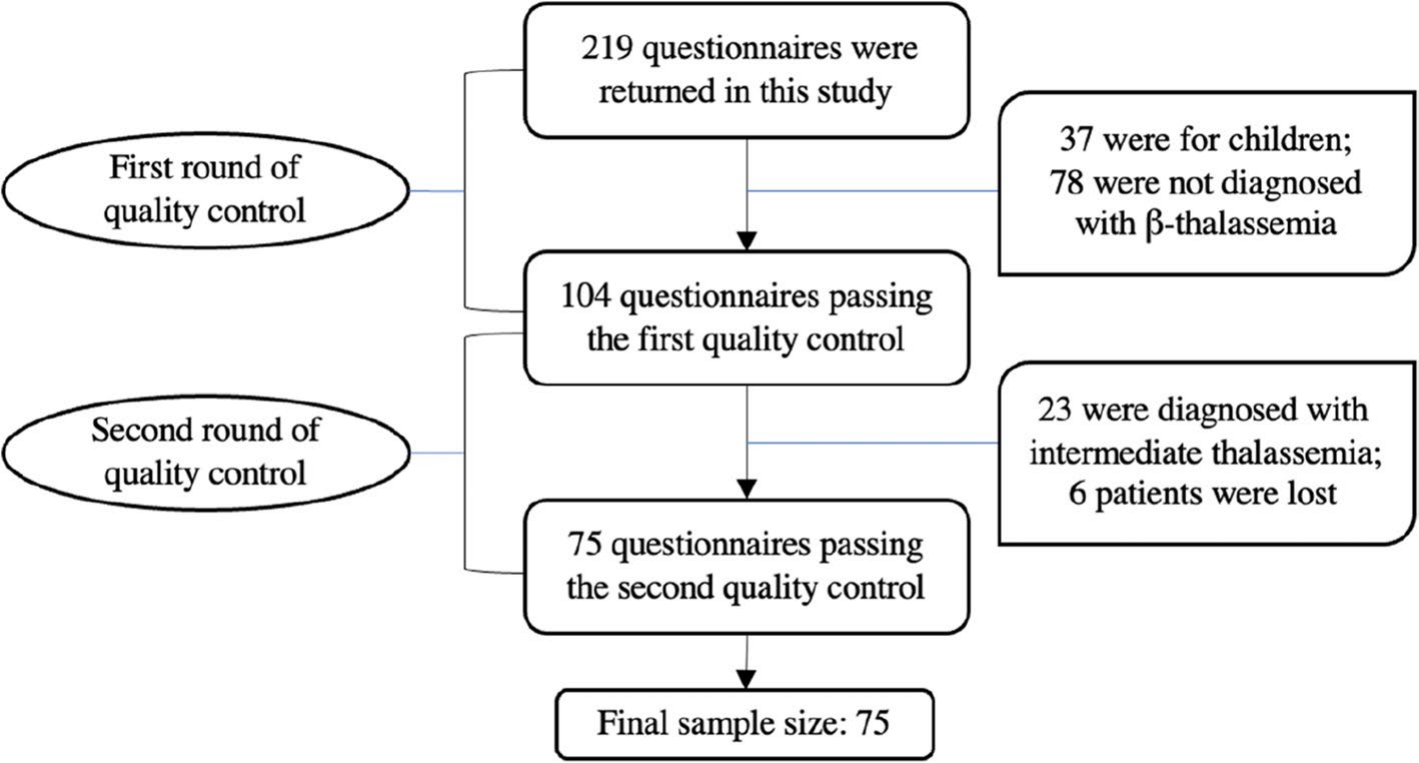
The process of the two-round of quality control

We trained all investigators before the study to unify the caliber of the study, the way of interviewing and the way of filling out the questionnaires. After collecting the questionnaires, in order to ensure the accuracy, credibility and reliability of the results of the questionnaire feedback, we conducted two rounds of strict quality control checks on the completion of the questionnaire by one-to-one telephone interviews to avoid omissions, logical errors and irregularities. Figure 1 illustrated the specific process of the two-round of quality control.

According to the guidelines for the diagnosis and treatment of β-TM (2017) [24], developed by the Blood Group of the Chinese Medical Association and Pediatrics Branch, 75 adult patients diagnosed with β-TM and a primary informal caregiver of each patient were included in the study. Inclusion criteria for adult patients were age ≥ 18 years old and a diagnosis of β-TM before the study. In addition, the patient and a primary informal caregiver had to understand the content of the questionnaire, be familiar with the entire treatment process, and complete two quality interviews.

### Data collection

The standardized questionnaire including a patient section and an informal caregiver section, was self-reported by eligible adult patients with β-TM and their primary informal caregivers. We extracted patient demographics (sex, age, education level, employment status, total medical cost, comorbidities, interruption of blood transfusion therapy or iron chelation therapy, and social support), and informal caregiver demographics (sex, age, marital status, education level, annual household income, the days of monthly caregiving, help from other caregivers, care for others and caregiver burden) from the questionnaire.

Annual household income and total medical cost were extracted from the online survey. Total medical cost comprising the costs for blood transfusion therapy, iron chelation therapy and adverse reaction therapy since diagnosis to date, included out-of- pocket payments (by patients themselves) and payments covered by health insurers.

Caregiver burden was measured using the Chinese version of the Zarit burden interview (ZBI) and filled out by caregivers. The ZBI was designed by Zarit and adapted by Lu et al. [25]. This Chinese version contains 22 items with two dimensions, including 12 entries for personal burden and 6 entries for role burden [26]. Each item is rated on a five-point Likert scale (never: 0; rarely: 1; sometimes: 2; quite frequently: 3; and always: 4) and summed to generate a caregiver burden score ranging between 0 and 88. Higher scores indicate a higher burden. Burden was differentiated into three levels: mild (< 21), moderate (21–40), and severe (> 40) [27]. The Chinese version of the ZBI has been widely used in China (Cronbach α = 0.921) [28].

Social support in adult patients was measured using the Chinese version of the social support rating scale (SSRS) compiled by Xiao Shuiyuan in 1994 [29], and filled out by patients. The SSRS is a 10-item scale that consists of three dimensions (objective support, subjective support, and utilization). Objective support is independent of the individual’s feelings and exists objectively, including direct material assistance and the existence and participation in social networks and group relationships, such as stable (family, marriage, friends, colleagues, etc.) or unstable (informal groups, temporary social interactions) social connections; subjective support refers to an individual’s emotional experience and satisfaction of being respected, supported, and understood in society; utilization of social support refers to the extent to which the individual utilizes the above objective and subjective social resources [29]. The total score ranges from 12 to 66, with a higher score indicating a higher level of social support. The SSRS has been used in a wide range of studies in the Chinese population (Cronbach α = 0.724) [30].

### Statistical analysis

Statistical analyses were performed using IBM SPSS Statistics (SPSS version 26.0, Inc., Chicago, IL, USA). Descriptive statistics were used to report the characteristics (demographic and clinical) and study variables of the adult patients and their informal caregivers. Categorical variables are presented as frequencies (n) and percentages (%), while continuous variables are presented as median, means and standard deviations (mean ± SD). Independent t-tests were conducted to evaluate the effect of characteristics (demographic and clinical) on the caregiver burden. Spearman correlation analyses were utilized to examine the correlation between caregiver burden and continuous variables (three dimensions of social support, age of patients and informal caregivers, total medical cost, monthly caregiving and annual household income). Based on evidence from previous studies [15, 31, 32], we hypothesized that patients’ age, history of interruption of blood transfusion and iron chelation therapy, and social support, as well as informal caregivers’ age, marital status, monthly caregiving, and taking care of others would be potential predictors of caregiver burden. Therefore, they were included in multiple linear regression. The 95% confidence intervals were computed, with *p*-values < 0.05 considered statistically significant. The 95% confidence intervals were computed, with *p*-values < 0.05 considered statistically significant and *p*-values < 0.1 considered statistically borderline significant [33].

### Ethical considerations

The ethics committee of the Centre for Health Management and Policy Research, Shandong University, approved the ethical application of this study on November 1, 2021 (approval no. ECSHCMSDU20211101). Before the investigation, we informed the participants of the study purpose and process and obtained their informed consent.

## Results

Seventy-five adult patients with β-TM and 75 informal caregivers of the patients completed the questionnaire. The demographics of the adult patients and their informal caregivers are presented in Table 1. Among the patients, approximately half (50.7%) were men, with a mean age of 24.69 years. Most patients (66.7%) had comorbidities, nearly half (56%) of whom had a history of interrupted blood transfusion therapy, and over half (61.3%) had a history of interrupted iron chelation therapy. The average score of patients on social support was 30.57 ± 5.54. The informal caregivers were mostly women (74.7%), with a mean age of 50.60 years. Nearly three-quarters (73.3%) of informal caregivers were married, and more than a half (58.7%) had an education level of middle school or below. The mean monthly caregiving was 12.59 days, most (70.7%) of the informal caregivers took care of patients without help from others, and most (62.7%) also cared for other people at the same time.

**Table 1:** Basic characteristics of adult patients with β-thalassemia major and their informal caregivers (*n* = 75)

Variables	N/Median(Mean ± SD)	Percent (%)
**Patient**		
Sex		
Female	37	49.3
Male	38	50.7
Age (years)	22(24.69 ± 5.59)	
≤ 22	37	50.7
> 22	38	49.3
Education level		
High school and below	37	49.3
Above high school	38	50.7
Full-time job		
No	49	65.3
Yes	26	34.7
Total medical cost (￥)	810,000(987,506.32 ± 784,960.41)	
≤ 810,000	38	49.3
> 810,000	37	50.7
Comorbidities		
No	25	33.3
Yes	50	66.7
Interruption of blood transfusion therapy		
No	42	56
Yes	33	44
Interruption of iron chelation therapy		
No	46	61.3
Yes	29	38.7
Social support	30(30.57 ± 5.54)	
Objective support (points)	6(6.43 ± 1.62)	
≤ 6	41	54.7
> 6	34	45.3
Subjective support (points)	17(17.84 ± 4.45)	
≤ 17	39	52
> 17	36	48
Utilization (points)	6(6.31 ± 1.74)	
≤ 6	44	58.7
> 6	31	41.3
**Informal caregiver**		
Sex		
Female	56	74.7
Male	19	25.3
Age (years)	51(50.60 ± 9.16)	
≤ 51	38	49.3
> 51	37	50.7
Marital status		
Single	20	26.7
Married	55	73.3
Education level		
Middle school and below	44	58.7
Above middle school	31	41.3
Annual household income (￥)	60,000(84,005.33 ± 101,213.21)	
≤ 60,000	38	49.3
> 60,000	37	50.7
Monthly caregiving (days)	7(12.59 ± 11.58)	
≤ 7	39	52
> 7	36	48
With the help of other caregivers		
No	53	70.7
Yes	22	29.3
Caring for others		
No	28	37.3
Yes	47	62.7

*SD* Standard deviation

The mean ZBI total score was 38.00 ± 17.02, which was medium burden, and the score range was from 0 to 83. The average scores of personal burden and role burden were 20.15 ± 9.08 and 9.44 ± 5.58, respectively. Severe caregiver burden was present in a total of 38.67% of patient informal caregivers (Table 2).

**Table 2:** Total and dimensional scores of ZBI for primary informal caregivers of adult patients with β-thalassemia (*n* = 75)

Itmes	N (%)	Mean ± SD	Minimum	Maximum
Personal burden	75(100)	20.15 ± 9.08	0	43
Role burden	75(100)	9.44 ± 5.58	0	24
ZBI total score	75(100)	38 ± 17.02	0	83
Mild burden	11(14.67)	11.91 ± 6.41	0	20
Moderate burden	35(46.67)	32.14 ± 5.27	22	40
Severe burden	29(38.67)	54.97 ± 10.42	42	83

*SD* Standard deviation

The univariate analysis reported a higher caregiver burden among caregiver of patients who had interrupted blood transfusion therapy (*p* < 0.05). Additionally, informal caregivers who needed to care for others bore a heavier caregiving burden compared to that in informal caregivers caring only for patients with *β-TM* (*p* < 0.05) (Table 3).

**Table 3:** Differences in caregiver burden scores across subgroups for the characteristics of patients and informal caregivers (*n* = 75)

Variables	Caregiver burden Mean ± SD	t	*P*-value
**Patient**			
Sex		-0.799	0.427
Female	36.41 ± 16.36		
Male	39.55 ± 17.72		
Age (years)	38.00 ± 17.02	1.239	0.219
≤ 22	40.39 ± 17.18		
> 22	35.54 ± 16.73		
Education level		1.073	0.287
High school and below	40.14 ± 17.39		
Above high school	35.92 ± 16.61		
Full-time job		1.114	0.269
No	39.59 ± 17.22		
Yes	35 ± 16.55		
Total medical cost (￥)	38.00 ± 17.02	1.128	0.263
≤ 810,000	40.18 ± 15.95		
> 810,000	35.76 ± 18.01		
Comorbidities		-0.257	0.798
No	37.28 ± 18.45		
Yes	38.36 ± 16.44		
Interruption of blood transfusion therapy		-2.052	**0.044**
No	34.5 ± 16.55		
Yes	42.45 ± 16.8		
Interruption of iron chelation therapy		-1.417	0.161
No	35.8 ± 17.85		
Yes	41.48 ± 15.26		
Social support	38.00 ± 17.02		
Objective support (points)	38.00 ± 17.02	0.407	0.686
≤ 6	38.73 ± 14.94		
> 6	37.12 ± 19.43		
Subjective support (points)	38.00 ± 17.02	-0.527	0.601
≤ 17	37.00 ± 16.44		
> 17	39.08 ± 17.79		
Utilization (points)	38.00 ± 17.02	-0.342	0.733
≤ 6	37.43 ± 16.77		
> 6	38.83 ± 17.62		
**Informal caregiver**			
Sex		-1.381	0.171
Female	36.43 ± 17.74		
Male	42.63 ± 14.08		
Age (years)	38.00 ± 17.02	0.364	0.717
≤ 51	38.71 ± 19.23		
> 51	37.27 ± 14.64		
Marital status		0.649	0.522
Single	40.6 ± 22.82		
Married	37.05 ± 14.5		
Education level		0.000	1.000
Middle school and below	38 ± 16.44		
Above middle school	38 ± 18.08		
Annual household income (￥)	38.00 ± 17.02	1.393	0.168
≤ 60,000	40.68 ± 18.19		
> 60,000	35.24 ± 15.49		
Monthly caregiving (days)	38.00 ± 17.02	-1.492	0.140
≤ 7	35.21 ± 16.72		
> 7	41.03 ± 17.05		
With the help of other caregivers		0.089	0.929
No	38.11 ± 17.84		
Yes	37.73 ± 15.26		
Caring for others		-2.512	**0.014**
No	31.82 ± 16.14		
Yes	41.68 ± 16.61		

*SD* Standard deviation

Correlation between continuous variables and caregiver burden are shown in Table 4. The Spearman correlation coefficient between caregiver burden and monthly caregiving was significantly positive (*p* < 0.05).

**Table 4:** Spearman correlation analysis results between caregiver burden and selected features (*n* = 75)

Variable	1	2	3	4	5	6	7	8
1. Caregiver burden	-	-	-	-	-	-	-	-
2. Objective support (points)	-0.078	-	-	-	-	-	-	-
3. Subjective support (points)	-0.013	0.178	-	-	-	-	-	-
4. Utilization(points)	-0.029	-0.030	.237^a^	-	-	-	-	-
5. Patient’s Age(years)	-0.153	-0.057	-.290^a^	-0.201	-	-	-	-
6. Total medical cost (￥)	-0.076	0.002	-0.013	-0.025	-0.045	-	-	-
7. Informal caregiver’s age (years)	0.018	-0.195	-.326^b^	-0.053	.461^b^	-0.008	-	-
8. Monthly caregiving (days)	**0.265**^**a**^	-0.099	-0.183	-0.120	0.061	0.018	.241^a^	-
9. Annual household income (￥)	-0.081	0.035	-0.042	-0.164	-0.022	0.012	-0.006	0.039

^a^Correlation is significant at the 0.05 level (2-tailed)

^b^Correlation is significant at the 0.01 level (2-tailed)

In the multiple linear regression model, the significant factors associated with caregiver burden included interruption of blood transfusion therapy, taking care of others, and informal caregiver’s age and marital status. Patients with a history of interrupted blood transfusion therapy and informal caregivers who needed to care for others were positively associated with caregiver burden (*p* < 0.05). Age of informal caregivers were significantly positively associated with caregiver burden (*P* < 0.1). Married informal caregiver status was negatively correlated with caregiver burden (*p* < 0.05). The model showed a good fit (*R*^2^ = 0.26) and multicollinearity was not observed (tolerance > 0.2, VIF < 10) (Table 5).

**Table 5:** Multiple linear regression summary of predictors for caregiver burden (*n* = 75)

Variables	B^a^	Beta^b^	*P*-value	95.0%CI (Lower)	95.0%CI (Upper)
**Patient**					
Age(years)	0.321	0.173	0.204	-0.178	0.819
Interrupt blood transfusion therapy	9.509	0.279	**0.027**	1.107	17.910
Interrupt iron chelation therapy	3.121	-0.090	0.452	-5.122	11.363
Objective support(points)	-1.689	-0.161	0.164	-4.087	0.709
Subjective support(points)	0.268	0.070	0.565	-0.659	1.196
Utilization(points)	-0.975	-0.100	0.390	-3.226	1.276
**Informal caregiver**					
Age(years)	-0.650	-0.213	**0.078**	-1.376	0.076
Married	-10.396	-0.272	**0.034**	-19.972	-0.820
Monthly caregiving(days)	0.048	0.033	0.793	-0.314	0.410
Take care of others	11.141	0.319	**0.006**	3.258	19.024

*CI* Confidence interval

^a^ Unstandardized beta

^b^ Standardized beta

## Discussion

This study is the first nationwide survey of the caregiver burden of adult patients with *β-TM* in mainland China, with a focus on high-incidence areas. In this study, the caregiver burden of adult patients with *β-TM* was predominantly moderate-to-severe (38.00 ± 17.02), similar to the findings of studies on caregiver burden in children with *β*-thalassemia in India and Pakistan [23, 34], and higher than those in some studies on adult patients with cancer (34.2 ± 16.4) [35], but lower than some studies on adult patients undergoing hematopoietic stem cell transplantation (45.6 ± 13.3) [32].

The mean age of patients with *β-TM* in this study was only 24.7 ± 5.6 years, which aligned to the literature reporting that the highest life expectancy of patients with *β-TM* in Guangxi, China, was only 28 years [10]. And it indicated that because they usually had poor quality of life, short life expectancy, and high mortality [36]. However, most patients with *β-TM* in the United Kingdom live at least until their mid-forties. This may be because about half of the patients in the UK with *β-TM* are treated with iron chelation therapy through daily intramuscular injection of desferrioxamine, administered nightly as an 8–12 h subcutaneous infusion, for at least 5 nights per week. This became standard management in 1982 and has been shown to improve survival [9].

Regarding the social support level for patients with *β*-thalassemia, we observed low level scores on the SSRS and its subdimensions, which is in alignment with many other studies [14, 37]. Compared to patients with other diseases, complications associated with blood transfusion and iron chelation therapies can lead to physical problems such as facial bone deformities, growth retardation, and psychological problems [38, 39], resulting in a lack of social acceptance. Therefore, patients may be unwilling to tell others about their experiences to receive help. Other studies have also shown that adult patients with *β*-thalassemia have less social support, and family is the most important source, while friends are the least important source of social support [37].

Among the informal caregivers, more than 70% were married women, aged around 50 years old, and more than three-fifths cared for patients without help from others, and also needed to take care of others besides adult patients with *β-TM*. A study conducted in Rawalpindi reported similar result [34]. Males are expected to be breadwinners in familial and societal contexts, and because of their unique physical and psychological characteristics, females are regarded as better caregivers than males. Females can not only maintain a good rehabilitation environment but also coordinate conflicts [40]. Therefore, females are left to care for sick children at home, and they also follow the traditional labor division in households.

We found that patients who had interrupted their blood transfusion therapy were positively associated with caregiver burden; that is to say, patients who had interruptions in their blood transfusion therapy their caregivers tend to have a higher level of burden. This was consistent with the results of patient and caregiver burden of transfusion-dependent *β*-thalassemia [41]. Abbas et al. also presented in a qualitative study that patients’ noncompliance with medication regimens can impose severe stress on informal caregivers [42]. One possible explanation for this phenomenon is that adult patients exceed the best age for hematopoietic stem cell transplantation, which is the only cure for patients with thalassemia; thus, they are subject to lifelong blood transfusion [43]. However, lifelong blood transfusion can lead to iron overload and increase the risk of death. Some experts have pointed out that only long-term blood transfusion therapy combined with iron chelator therapy can allow adult patients with *β-TM* to survive for a long time [44]. Although interrupting blood transfusion therapy can save some medical costs in the short-term, once complications arise and the condition worsens, surgical treatment is required, and patients may lose their labor capacity. In addition, the cost of follow-up treatment greatly increases the financial burden on the family, causing the caregiver to experience both physical and mental pressures [45].

We also observed a positive association between older informal caregivers and caregiver burden, and between informal caregivers who needed to take care of others and caregiver burden. This result is consistent with those of another study on the burden among informal caregivers of patients with acute leukemia and epilepsy [31]. Most informal caregivers of adult patients with thalassemia are > 50 years of age; thus, they are in the middle-aged or elderly stage of the disease-prone period. Informal caregivers also need to take care of other people besides the patients, such as their own parents, children, and grandchildren. The long-term doubling of time, money, and energy consumption greatly increases the caregiver burden, which, in turn, affects their physical and mental health and may lead to anxiety, depression, and other emotions [46], thus further increasing the caregiver burden.

In addition, we identified a negative association between married informal caregiver status and caregiver burden, which is consistent with the results related to caregiver burden in patients undergoing hemodialysis [47]. The caregiver burden of single informal caregivers was higher than that of married informal caregivers. This may be because the family function of married informal caregivers is better; informal caregivers can obtain more resources from family members, such as psychological and financial support, to cope with the patient’s disease, and the family can also help the patient build confidence in the recovery, thereby reducing caregiver burden.

This study also showed a positive association between monthly caregiving and caregiver burden, the longer care, the higher caregiver burden. The results of a study on hemodialyzers in Iran similarly revealed that the burden of care increases with the amount of time caregivers provide care [48]. Also, the findings of studies in Western countries have found that the longer time the caregiver spends with the patient was found to be a predictor of greater burden experienced by the caregivers, and by increased duration of disease, the primary caregiver experienced greater burden. Because burnout syndrome develops in family members, particularly in primary informal caregivers [49, 50]. As a study has revealed, worse financial situation of caregivers is a major cause of higher caregiver burden [51]. The informal caregivers were particularly likely to be absent from work or unemployed due to more monthly caregiving, leading to worse financial circumstances and heavier care burden [52]. Therefore, these determinants require consideration when seeking to alleviate caregiver burden.

### Strengths and limitations

This study contributes to the literature in several ways. First, most studies related to thalassemia in mainland China are clinical studies, and less attention has been paid to the determinants of caregiver burden, which were emphasized in the present study. Second, existing studies on the caregiver burden of patients with thalassemia have mainly focused on pediatric patients, ignoring adult patients who have relatively worse quality of life, higher mortality, and more serious complications due to long-term irregular treatment. This study attempted to address this gap. Finally, the findings of this study provide a reference to identify informal caregivers with a higher burden among those caring for patients with β-TM.

This study has several limitations. First, due to the difficulty in recruiting samples, the sample size in some provinces was relatively small, which limited the representativeness and generalizability of the conclusions. Nevertheless, sample size of adult patients with major β-thalassemia in this study was relatively representative since thalassemia is a rare disease, β-thalassemia is only one of the three principal types, and surviving adult patients with major β-thalassemia are an even smaller group. Second, the snowball sampling was used in this study, voluntary participating respondents probably represented patients who were more compliant and proactive in general. Therefore, the results of the study may be biased to a degree that requires cautions when generalizing to the entire patient population, and future validation in larger samples will be necessary. Third, this study was based on a cross-sectional design; therefore, it could only test the associations among variables simultaneously, rather than the causal relationships. Therefore, future large-scale (e. g., adult patients with *β*-thalassemia intermediate and their informal caregivers) and prospective cohort studies should be considered.

### Practical, educational, and research implications

This study is the first to investigate the determinants of burden among informal caregivers of adults with β-TM in mainland China, providing evidence-based insights to assist policymakers in identifying burden factors. These results emphasized to minimize the burden of informal caregivers of thalassemia patients in China, which will assist the relevant parts of the government to formulate effective strategic planning to optimize the treatment of patients’ security and reduce social discrimination. The study will also be useful for medical professionals and nursing educators to establish effective strategies, such as strengthening grassroots training, conducting scientific education campaigns, and revising caregiver needs assessments, in order to enhance the quality of care and the implementation of home-based care.

## Conclusions

The present study demonstrated that the informal caregivers of adult patients with *β-TM* in mainland China experienced a moderate-to-severe caregiver burden. The informal caregivers experienced a higher care burden when the patients had interrupted their blood transfusion therapy or the informal caregivers who were older or needed to take care of others. Additionally, married informal caregivers experienced less burden compared to that in informal caregivers who were not married. Therefore, the relevant government departments should make efforts to ensure that patients with thalassemia receive regular blood transfusion and iron chelation therapies by improving blood and drug availability. In addition, society should pay more attention to adult patients with thalassemia. Moreover, further increased social support for the informal caregivers of patients with thalassemia is required from society, relatives, and friends.

## Data Availability

The datasets used and/or analysed during the current study are available from the corresponding author on reasonable request.

## Abbreviations

β-TM: β-thalassemia major
COVID-19: Coronavirus
ZBI: Zarit burden interview
SSRS: Social support rating scale
SD: Standard deviation
CI: Confidence interval
